# ID 87 - Análise de Efetividade e Segurança do Uso de Levosimendana em Pacientes com Insuficiência Cardíaca Descompensada

**DOI:** 10.5327/2237-9622.2025.v34s1.87

**Published:** 2025-11-25

**Authors:** Erika Michelle do Nascimento Facundes Barbosa, Bárbara Helena de Brito Ângelo, Lúcia Reis do Nascimento, Giselle de Souza Paiva, Nara Gualberto Cavalcanti, Isabella Valois Pedrosa Porto, Fabiana Lícia Araújo dos Santos

**Keywords:** levosimendana, Avaliação de Tecnologias em Saúde, eficácia, insuficiência cardíaca

## Abstract

**Introdução:**

A insuficiência cardíaca descompensada (ICD) é uma condição crítica responsável por elevadas taxas de hospitalização e mortalidade em todo o mundo, representando um desafio significativo para os sistemas de saúde. Nesse contexto, a levosimendana, um agente inotrópico e vasodilatador, tem sido amplamente utilizado para o manejo de pacientes em estado agudo. Os objetivos deste trabalho foram identificar, avaliar e sumarizar as melhores evidências científicas disponíveis sobre a eficácia e a segurança de levosimendana como um medicamento da descompensação aguda da insuficiência cardíaca crônica.

**Método:**

A metodologia adotada envolveu a elaboração de Nota Técnica de Resposta Rápida (NTRR) com a seguinte pergunta de pesquisa: Qual a efetividade e segurança do uso de levosimendana em pacientes com insuficiência cardíaca descompensada? Foi realizada uma busca de evidências nas bases de dados PubMed, LILACS e Google utilizando os seguintes descritores: "levosimendan", "dobutamine", "decompensated heart failure". Os critérios de inclusão foram ensaios clínicos randomizados e revisões sistemáticas com ou sem metanálise, com pelo menos um desfecho de interesse.

**Resultados:**

Foram incluídos para análise três ensaios clínicos randomizados (ECR), totalizando uma amostra de 691 pacientes, uma revisão sistemática com metanálise (3.052 pacientes de 22 ECR) e um estudo de custo. Também foi feita pesquisa de relatório de recomendação ou diretrizes da Comissão Nacional de Incorporação de Tecnologias do Ministério da Saúde(Conitec) sobre o assunto e nenhuma evidência foi localizada. Os ECR analisados mostraram que o uso da levosimendana resultou em melhora nos desfechos de hospitalização por insuficiência cardíaca, débito cardíaco e parâmetros hemodinâmicos, quando comparado a outros tratamentos. Contudo, o uso do medicamento foi associado a uma maior incidência de hipotensão e arritmias cardíacas e, em um dos estudos, a um aumento numérico no risco de morte (49 de 350 num regime de levosimendana vs. 40 de 350 num regime de placebo aos 90 dias, p = 0,29). A revisão sistemática destacou os benefícios da levosimendana em subpopulações específicas, como pacientes em pós-operatório de cirurgia cardíaca, com insuficiência cardíaca isquêmica ou em terapia concomitante com β-bloqueadores. Em termos de custos, os gastos com medicamentos foram maiores no grupo de levosimendana, mas os custos diretos e indiretos com internamento na terapia intensiva foram menores.

**Conclusão:**

Apesar dos benefícios supracitados, a literatura reconhece risco de eventos cardiovasculares adversos em pacientes em uso de levosimendana. O único estudo recuperado sobre os custos de pacientes em uso de dobutamina versus levosimendana apresenta diferença estatisticamente significativa a favor da levosimendana em se tratando de pacientes com internação em UTI. Diante do alto custo do fármaco e a incerteza de benefícios clínicos sobre desfechos clínicos relevantes, o uso de rotina em pacientes com IC descompensada no contexto de um hospital geral do SUS não se justificou. A NTRR recomendou, portanto, a construção de um protocolo de uso institucional, com definição clara do perfil de paciente a ser contemplado com esta terapia, bem como os parâmetros obrigatórios para iniciar o uso da levosimendana na terapia intensiva.

